# CHD5 inhibits metastasis of neuroblastoma

**DOI:** 10.1038/s41388-021-02081-0

**Published:** 2021-11-17

**Authors:** Astrid K. Laut, Carmen Dorneburg, Axel Fürstberger, Thomas F. E. Barth, Hans A. Kestler, Klaus-Michael Debatin, Christian Beltinger

**Affiliations:** 1 Section Experimental Pediatric Oncology, Dept. of Pediatrics and Adolescent Medicine, University Medical Center Ulm, Ulm, Germany; 2 Institute of Medical Systems Biology, Ulm University, Ulm, Germany; 3 Dept. of Pathology, University Medical Center Ulm, Ulm, Germany; 4 Dept. of Pediatrics and Adolescent Medicine, University Medical Center Ulm, Ulm, Germany

**Keywords:** Embryonal neoplasms, Paediatric cancer

## Abstract

*CHD5*, a tumor suppressor at 1p36, is frequently lost or silenced in poor prognosis neuroblastoma (NB) and many adult cancers. The role of CHD5 in metastasis is unknown. We confirm that low expression of CHD5 is associated with stage 4 NB. Forced expression of CHD5 in NB cell lines with 1p loss inhibited key aspects of the metastatic cascade in vitro: anchorage-independent growth, migration, and invasion. In vivo, formation of bone marrow and liver metastases developing from intravenously injected NB cells was delayed and decreased by forced CHD5 expression. Genome-wide mRNA sequencing revealed reduction of genes and gene sets associated with metastasis when CHD5 was overexpressed. Known metastasis-suppressing genes preferentially upregulated in CHD5-overexpressing NB cells included *PLCL1*. In patient NB, low expression of PLCL1was associated with metastatic disease and poor survival. Knockdown of PLCL1 and of p53 in IMR5 NB cells overexpressing CHD5 reversed CHD5-induced inhibition of invasion and migration in vitro. In summary, CHD5 is a metastasis suppressor in NB.

## Introduction

Neuroblastoma (NB) is the most common extracranial solid tumor of childhood. Half of the patients harbor metastases and survival of these high-risk patients remains poor [1, 2]. NB metastasizes into lymph nodes, bone marrow, bone, liver, skin and, rarely, lung and brain [1, 2].

While the cellular and molecular principles of metastasis, one of the hallmarks of cancer [3], are becoming clearer [4], little is known about the metastatic cascade in NB. Steps in the metastatic sequence of NB include initial dissociation of NB cells from the primary tumor followed by intravasation [5–10], survival of the cells in the circulatory system, and extravasation into organs where the NB cells grow into a metastatic mass [5, 7, 11–14].

Loss of 1p and 11q, gain of 17q and *MYCN* amplification are associated with metastatic disease in NB [15]. 1p36 is frequently deleted in aggressive NB [16–25]. This chromosomal segment contains *CHD5* at 1p36.31 [26–28], amongst several other genes. In NB, as in many adult cancers with 1p loss, one *CHD5* allele is deleted while the second allele is transcriptionally silenced [29, 30]. Silencing occurs by methylation or other epigenetic mechanism and may involve miRNAs [31]. Deletion and silencing of *CHD5* cause low or absent expression of CHD5 in NB, associated with high-risk factors including advanced stage, and with low survival [29, 32]. CHD5 is a chromodomain-helicase-DNA-binding protein that forms a nucleosome remodeling and deacetylation (NuRD) complex acting on chromatin structure and gene expression [33–35].

*CHD*5 is a bona fide tumor suppressor gene in NB, as shown by attenuation of clonogenicity in vitro and decrease of tumorigenicity in mice when overexpressed [36]. This tumor suppressor function depends on a functional p19^ARF^–p53 axis, as determined by chromosome engineering in mice [37].

Since low CHD5 expression is associated with advanced stage, we reasoned that CHD5 may inhibit metastasis in NB. We now provide experimental evidence that CHD5 is indeed a metastasis suppressor in NB.

## Results

### In patient NB low CHD5 expression is associated with stage 4 metastatic disease and low survival

**Fig. 1:**
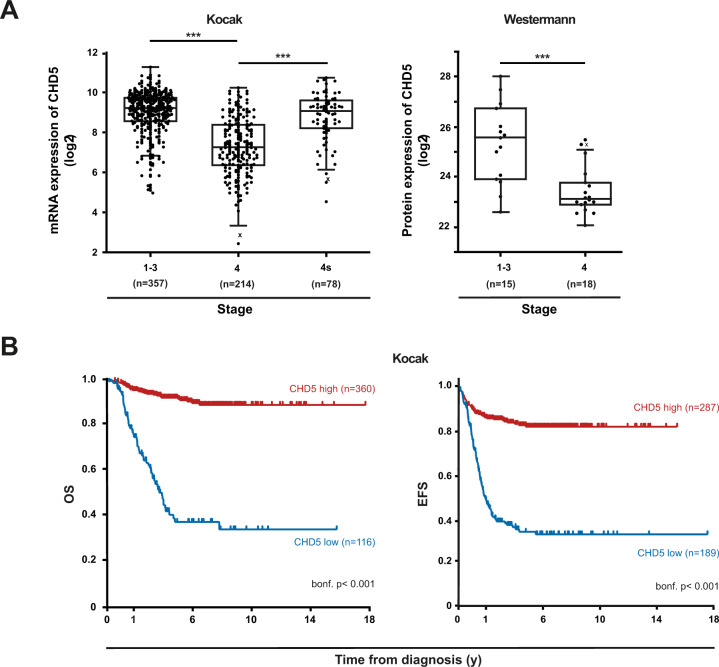
**Low expression of CHD5 is associated with aggressive, metastatic disease in patient NB.** CHD5 mRNA expression of 649 patient NB (Kocak-GSE45547) and CHD5 protein expression of 34 patient NB (Westermann-34-LFQ-fw2010prot) were analyzed using the R2 genomics analysis and visualization platform (http://r2.amc.nl). **A** Low CHD5 expression is associated with stage 4 metastatic disease. CHD5 mRNA expression (left panel) and protein expression (right panel) depending on stage are shown. One-way ANOVA was performed. ***p < 0.001. **B** Low CHD5 mRNA expression is associated with low overall and event-free survival. Kaplan–Meier survival estimates are shown for overall survival (OS) and event-free survival (EFS). Results of the log-rank test are indicated. The cut-off was determined using the scanning method.

To confirm and extend previous data of the association of CHD5 mRNA expression with metastasis of NB and patient survival [29, 36] we analyzed a large cohort (*n* = 649) of clinically annotated NB. Indeed, low CHD5 mRNA expression is significantly associated with stage 4, the most metastatic stage (Fig. 1A, left panel) and decreased survival (Fig. 1B). This association is not seen with stage 4s (Fig. 1A left panel). In a second, small cohort (*n* = 34) the association of CHD5 with stage 4 was also evident on the protein level (Fig. 1A, right panel).

### In NB cell lines with 1p loss CHD5 is a tumor suppressor that may depend on p53

**Fig. 2:**
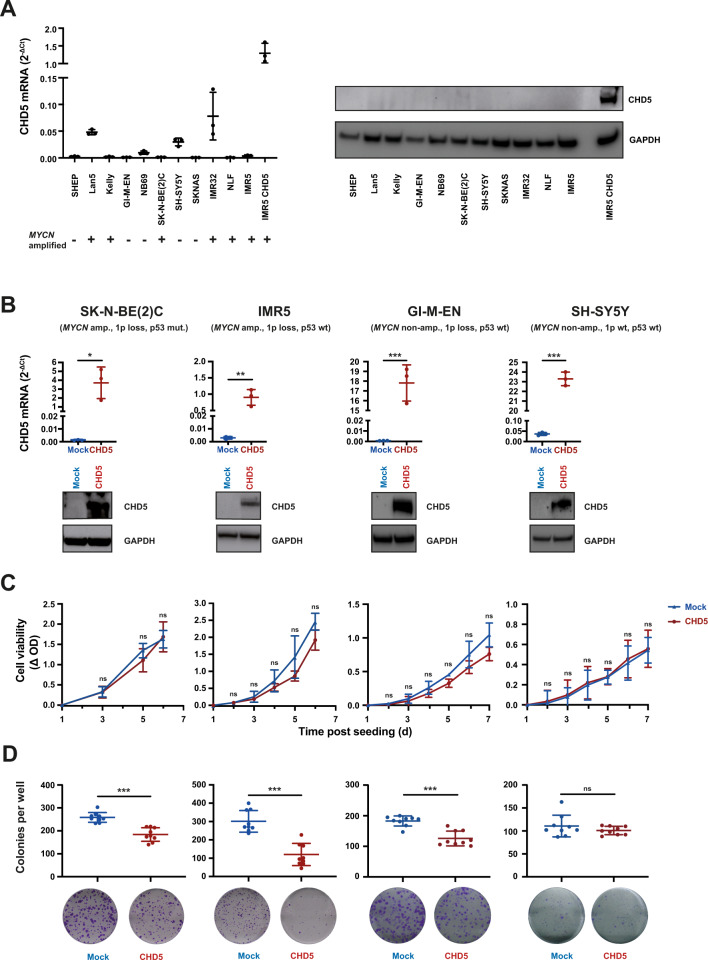
**CHD5 is a tumor suppressor in NB cell lines with 1p loss.** **A** NB cell lines express very low levels of CHD5. NB cells were subjected to qRT-PCR for CHD5 normalized to HPRT expression, MYCN status of the cell lines is indicated (left panel). Cells were investigated by Western Blot analysis for CHD5 protein with GAPDH as loading control (right panel). IMR5 cells with forced expression of CHD5 were used as positive control. **B** Forced expression of CHD5. SK-N-BE(2)C, IMR5, GI-M-EN and SH-SY5Y cells were stably transduced with either a CHD5 expression or a mock control construct. Cells were analyzed by qRT-PCR and Western Blot analysis for CHD5 expression. Shown is one representative experiment of three independent transductions. **C** Forced expression of CHD5 reduces cell viability of NB cells with 1p loss. Viable cells growing in 96-well plates were determined by OD measurements after MTT staining. Results are expressed as ΔOD of day 1. **D** Forced expression of CHD5 reduces clonogenic growth of NB cells with 1p loss. For clonogenic growth analysis, cells were stained with crystal violet 10 (SK-N-BE(2)C, IMR5), 11 (GI-M-EN), or 14 (SH-SY5Y) days after plating and colonies per well were counted. Shown is one representative experiment of three independent transductions. Means and SD are shown in (**A**–**D**). Statistical analysis was performed using the *t* test. ns not significant; *p < 0.05; **p < 0.01; ***p < 0.001. Experiments in (**B**–**D**) were repeated at least three times in triplicates, with similar results.

Next, we assessed CHD5 expression in a panel of 11 NB cell lines. CHD5 mRNA expression was very low, irrespective of *MYCN* amplification (Fig. 2A, left panel), in line with published data [26]. This corresponded with near-complete absence of CHD5 protein in the cell lines (Fig. 2A, right panel). To assess the effects of CHD5 on cell growth and clonogenicity, we transduced NB cells, which lack CHD5 expression, with either a CHD5 or a mock construct. For this we selected the *MYCN*-amplified cell lines SK-N-BE(2)C (1p loss, p53 non-functional), IMR5 (1p loss, p53 wt) as well as *MYCN* non-amplified cell lines GI-M-EN (1p loss, p53 wt) and SH-SY5Y (1p wt, p53 wt). CHD5 expression was detected at mRNA and protein levels (Fig. 2B). Forced CHD5 expression trended (without reaching statistical significance) to decrease cell growth of IMR5 and GI-M-EN cells, while growth of SK-N-BE(2)C and SH-SY5Y was not affected (Fig. 2C). Clonogenic growth was strongly decreased in IMR5 cells, less pronounced in SK-N-BE(2)C and GI-M-EN cells and unaltered in SH-SY5Y (Fig. 2C). Thus, inhibition of growth and clonogenicity was evident only or more pronounced, respectively, in the 1p deleted, p53 wildtype IMR5 and GI-M-EN cells. In summary, CHD5 is a tumor suppressor in NB cell lines with 1p loss that may depend on functioning p53.

### High expression of CHD5 in NB cells with 1p loss decreases proficiency of mechanisms important for metastasis

**Fig. 3:**
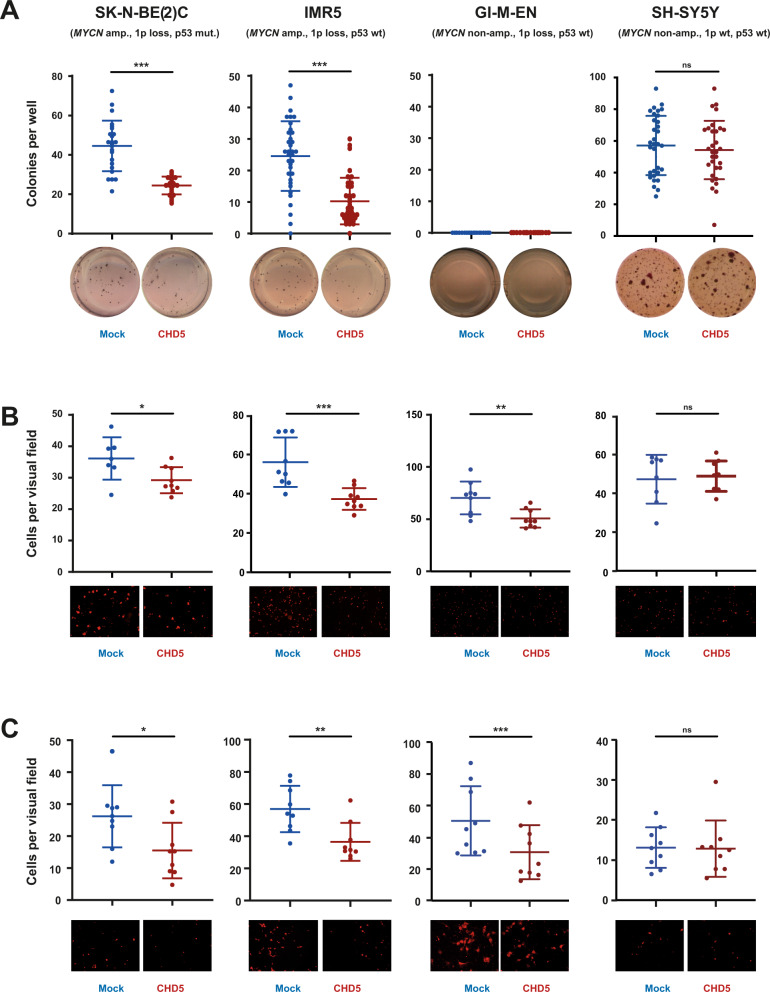
**Forced expression of CHD5 in NB cell lines with 1p loss impairs main steps of the metastatic cascade.** **A** Forced expression of CHD5 decreases anchorage-independent growth. SK-N-BE(2)C, IMR5, GI-M-EN and SH-SY5Y cells transduced with CHD5 expression or mock control constructs were seeded into soft agar in 24-well plates. MTT staining was used to visualize colonies after 12–13 days (IMR5, SK-N-BE(2)C), 8 weeks (SH-SY5Y), or 10 weeks (GI-M-EN). **B** Forced expression of CHD5 reduces adhesion to HUVECs. Transduced cells were labeled with DiI and seeded on a confluent monolayer of HUVECs. After 1 h, cells were fixed and adhering tumor cells per visual field (×10 magnification) were counted. **C** Forced expression of CHD5 reduces invasion and migration. Transduced cells were labeled with DiI and added to matrigel-coated transwell chambers. The top of the chamber contained medium with 1% serum and the lower part medium with 10% serum. 12 h (IMR5) or 20 h (SK-N-BE(2)C, GI-M-EN and SH-SY5Y) post seeding, cells that had migrated through the matrigel and membrane, and were attached to the lower surface of the membrane, were fixed. The number of invasive cells per visual field (×20 magnification) is depicted in the graph. Means and SD are shown in (**A**–**C**). Statistical analysis for (**A**–**C**) was performed using the *t* test. ns not significant; *p < 0.05; **p < 0.01; ***p < 0.001. Experiments were repeated at least three times, with similar results.

To address the question whether CHD5 functions as a metastasis suppressor in NB cells, the metastatic cascade was modeled in vitro. First, the ability of the cells to survive without cell-cell and cell-matrix contact (anoikis resistance), required for metastasizing cells in the blood stream, was investigated by challenging the cells to grow in low-density matrigel. IMR5 and SK-N-BE(2)C cells with forced CHD5 expression formed significantly less colonies when deprived of anchorage, while there was no effect of CHD5 overexpression in SH-SY5Y cells (Fig. 3A). GI-M-EN cells were unable to grow in soft agar (Fig. 3A). To assess the next major step of the hematogenous metastatic cascade, i.e., adherence of cancer cells to endothelium, adhesion of the NB cells to human umbilical vein endothelial cells (HUVECs) was determined. CHD5 overexpression decreased the capacity of cell lines with 1p loss to adhere to HUVECs, independent of *MYCN* status (Fig. 3B). To investigate the subsequent step in the metastatic cascade—invasion into and migration through the matrix of target organs—invasion and migration of the cells in matrigel was assessed. Forced CHD5 expression resulted in reduced capacity of cell lines with 1p loss to invade and migrate in extracellular matrix, while SH-SY5Y cells (1p wt) were unaffected (Fig. 3C). Taken together, high expression of CHD5 in the 1p-deleted SK-N-BE(2)C, IMR5 and GI-M-EN NB cells decreased their metastatic proficiency in vitro.

### Decreased homing of IMR5 cells with forced CHD5 expression

**Fig. 4:**
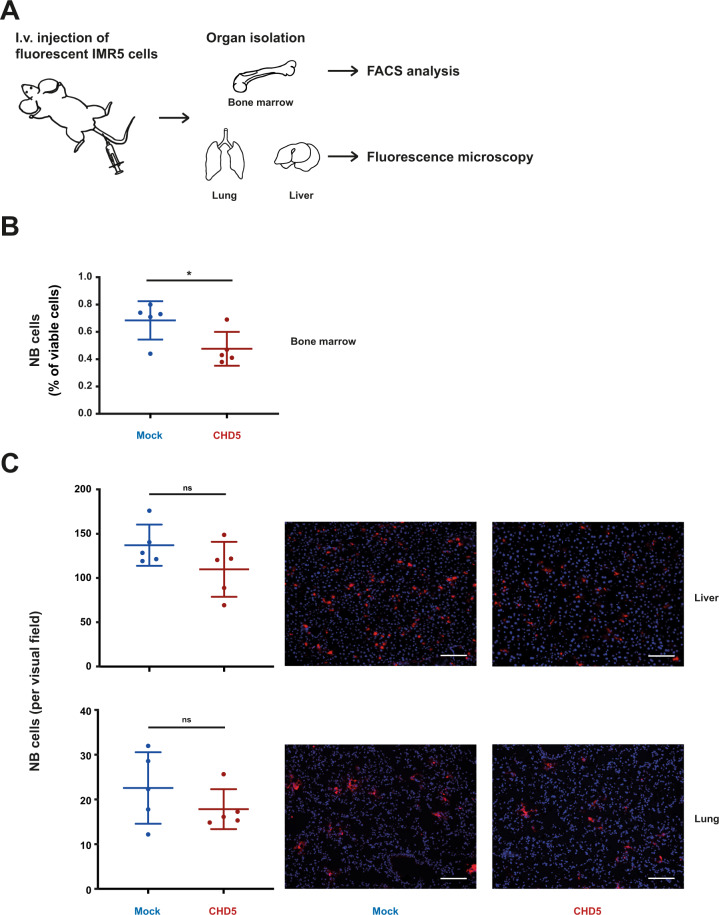
**Forced expression of CHD5 impairs homing of intravenously injected IMR5 cells.** **A** Schematic of the in vivo experiment to analyze homing of NB cells in mice. 5 × 10^6^ DiI-labeled IMR5 cells were injected into the tail vein of RAG2^−/−^cγc^−/−^ mice (five animals per group). 2 h after injection, mice were sacrificed. Bone marrow was subjected to FACS analysis, and liver and lung to fluorescence microscopy to determine the number of homed IMR5 cells. **B** Decreased homing of IMR5 with forced expression of CHD5 to bone marrow. Bone marrow of both femurs was extracted and the number of DiI-positive cells was determined using FACS analysis. **C** Decreased homing of IMR5 with forced expression of CHD5 to liver and lung. Lung and liver tissue were formalin-fixed, cryo-embedded, and subsequently counterstained with DAPI. The number of DiI-positive cells per visual field (30 fields per organ and mouse) is indicated. Scale bar equals 100 µm. Means and SD are shown for both panels. The *t* test was used for statistical analysis. ns not significant; *p < 0.05.

Given that CHD5 inhibited the metastatic cascade in vitro, we investigated its role in experimental metastasis of NB cells in vivo (Fig. 4A). To this end, homing of IMR5 cells was assessed. IMR5 cells with forced CHD5 expression homed significantly less to the bone marrow 2 h after i.v. injection (Fig. 4B) compared to mock-transduced cells, and trended (without reaching statistical significance) to home less into liver and lung (Fig. 4C). Interestingly, few cells homed to the lung, in line with the absence of lung metastases in NB patients (Fig. 4C). In summary, forced CHD5 expression reduced homing of IMR5 NB cells to bone marrow and, less so, liver, important metastasis sites in NB patients.

### Reduced metastatic burden by forced expression of CHD5

**Fig. 5:**
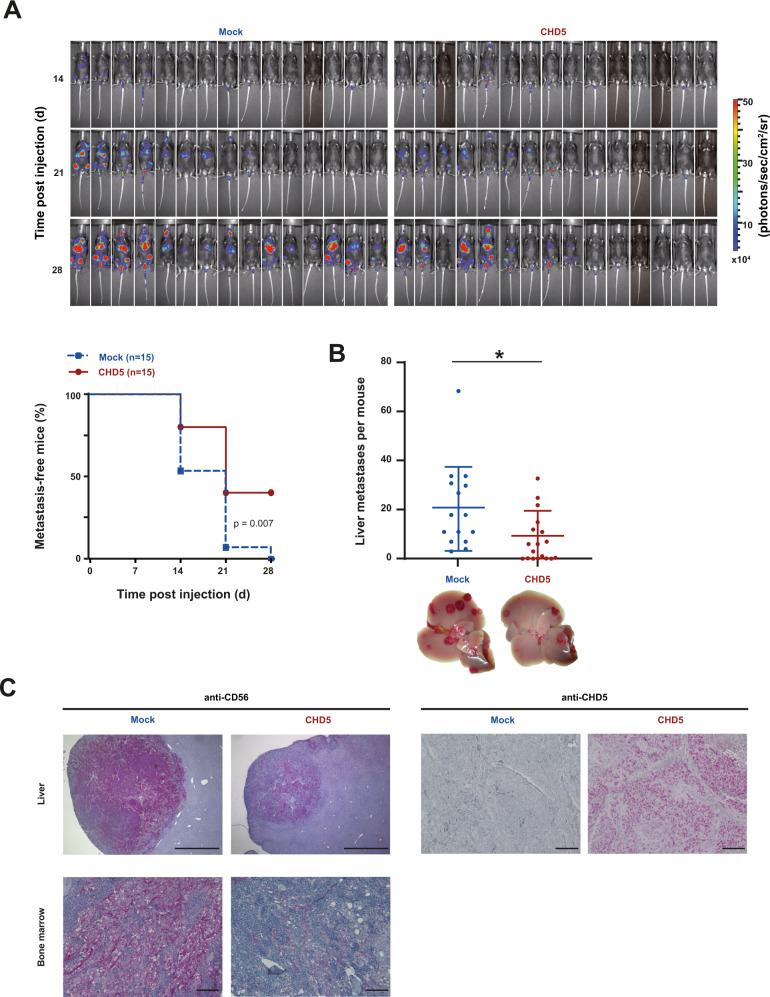
**Forced expression of CHD5 inhibits formation and growth of metastases.** **A** Forced expression of CHD5 increases latency and decreases incidence of metastases. 1 × 10^6^ transduced IMR5 cells stably expressing the firefly luciferase gene were injected into the tail vein of RAG2^−/−^cγc^−/−^ mice. Development of metastases was assessed once per week for 3 weeks by luminescence imaging following luciferin injection. The absence of metastases is depicted in the Kaplan–Meier plot. The log-rank test was used for statistical analysis. **B** Forced expression of CHD5 reduces the number of metastases. Mice were sacrificed 30 d post-tumor cell injection and organs were formalin-fixed. The number of metastases visible on the liver surface was determined. Means and SD are shown for each group. Statistical analysis was performed using the *t* test. *p < 0.05. **C** Immunohistochemistry confirms metastases and their persistent CHD5 overexpression. Formalin-fixed, paraffin-embedded liver and bone marrow samples from day 28 were subjected to immunohistochemistry for CD65, a neuroblastoma marker, and CHD5. Representative pictures for each group are shown. Scale bars equal 1 mm (liver) or 100 µm (bone marrow).

To investigate whether decreased homing of IMR5 cells with forced CHD5 expression translates into reduced metastatic burden in vivo, metastatic growth in mice was monitored for 4 weeks using bioluminescence in vivo imaging. Forced CHD5 expression decreased tumor incidence and moderately prolonged tumor latency (Fig. 5A). Furthermore, bioluminescent signals were less pronounced in mice injected with cells with forced CHD5 expression, indicating a lower metastatic burden (Fig. 5A upper panel). Mice were sacrificed 4 weeks post injection. Necropsy revealed less macroscopic liver metastases in mice that had received cells with forced CHD5 expression (Fig. 5B). Immunohistochemistry for CD56, a marker for NB cells, confirmed metastases in liver and bone marrow, while no difference in cell morphology or infiltrating growth was detected (Fig. 5C). Immunohistochemistry for CHD5 showed, that the CHD5 overexpression was maintained in the liver metastases (Fig. 5C). Proliferation (Supplementary Fig. 1A) and vascularization (Supplementary Fig. 1B) of the metastases were not altered by forced CHD5 expression. Taken together, these results show that CHD5 decreases the metastatic capacity of NB cells.

### GSEA suggests that CHD5 in NB cells is associated with an antimetastatic status

**Fig. 6:**
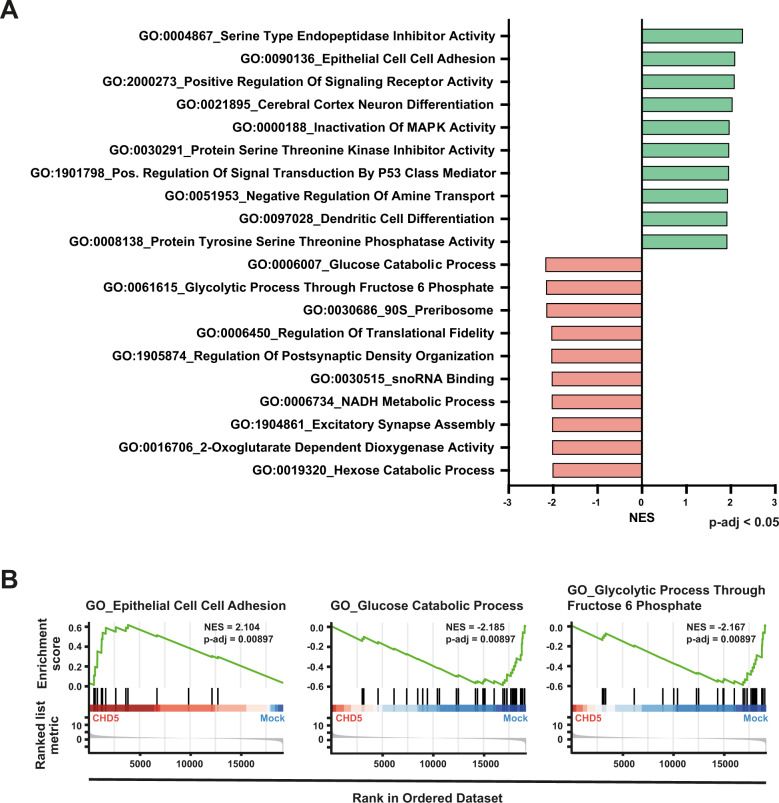
Gene sets indicative of an antimetastatic status are enriched in CHD5-overexpressing NB cells. **A** Enrichment and depletion of GO classes associated with antimetastatic mechanisms and metabolic flexibility, respectively. The transcriptomes of three independent sets of IMR5 cells overexpressing CHD5 and corresponding control cells were determined by mRNA sequencing. Gene set enrichment analysis (GSEA) was performed using the gene ontology (GO) classes. Results were filtered for p-adjusted and ranked according to the normalized enrichment score (NES) to identify the most relevant GO classes. The table shows the top ten significantly (*p* < 0.001) enriched (green) and depleted (red) GO classes. **B** Increased cell-cell adhesion and decreased glucose metabolism by CHD5 expression. GSEA plots are shown. Based on the ranked list metric, enrichment scores (green curves) and position of genes (black vertical lines) are shown. NES and p-adjusted values are shown.

To gain insights into molecular mechanisms mediating CHD5-dependent metastasis inhibition, transcriptome-wide mRNA sequencing followed by GSEA was performed. Upon CHD5 overexpression gene ontologies (GO) related to serine proteinase inhibition, cell-cell adhesion, neuronal differentiation, and inactivation of MAPK-signaling were among the top 10 of 98 significantly enriched gene sets compared to control cells (Fig. 6A). In contrast, gene sets related to glucose metabolism were predominantly enriched in control cells without detectable CHD5 expression compared to CHD5-overexpressing cells (Fig. 6B). These results imply that CHD5 expression in NB cells may be associated with an antimetastatic phenotype, including decreased metabolic flexibility.

### PLCL1 appears to contribute to the metastasis-inhibiting effect of CHD5 in vitro and in situ, and p53 in vitro

**Fig. 7:**
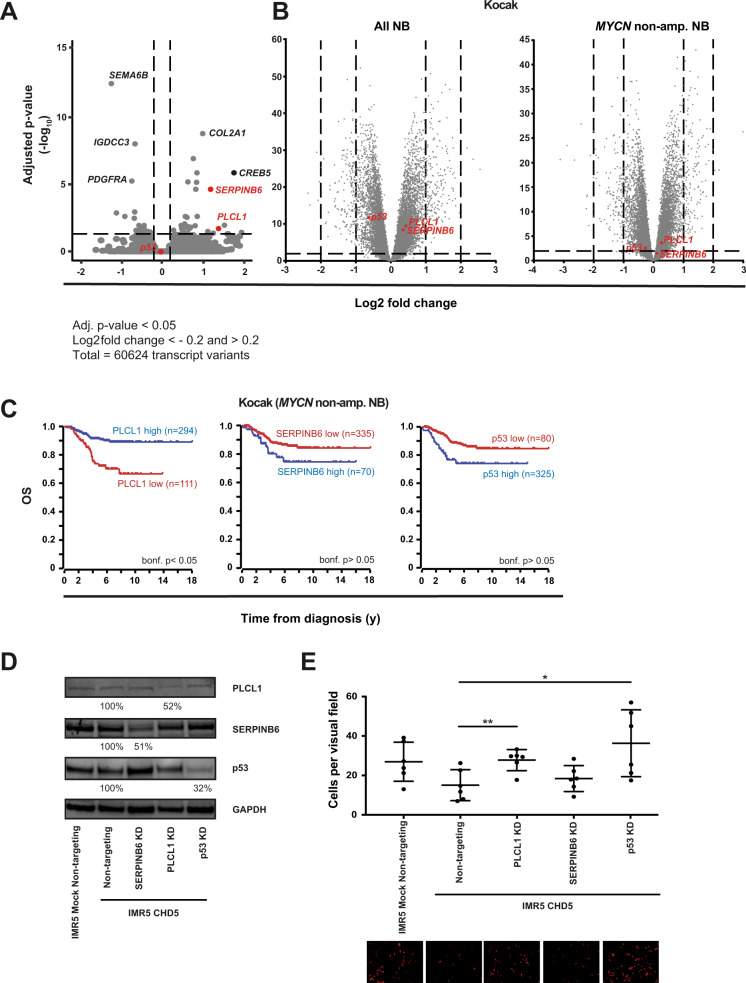
**PLCL1 contributes to the metastasis-inhibiting effect of CHD5in vitroandin situ, and p53in vitro.** **A** *PLCL1* and *SERPINB6* are among the top differentially expressed genes. The mRNA sequencing-derived transcriptomes of IMR5 cells overexpressing CHD5 and corresponding control cells were analyzed for differentially expressed genes. Gray and red dots in the volcano plot represent genes differentially expressed between both groups. **B** High PLCL1 expression in patient NB is associated with nonmetastatic disease independent of *MYCN* amplification. Gene expression depending on stage is shown in a clinically annotated NB dataset (Kocak, GSE45547, *n* = 571; R2 genomics analysis and visualization platform) for all NB of the dataset (left panel) or for *MYCN* non-amplified NB (right panel). Gray and red dots in the volcano plot represent genes differentially expressed between stage 1–3 versus stage 4 patients. **C** High PLCL1 expression is associated with increased overall survival. Kaplan–Meier overall survival (OS) estimates for expression of PLCL1, SERPINB6 and p53 in *MYCN* non-amplified NB of the Kocak dataset are shown. Results of the log-rank test are indicated. The cut-off was determined using the scanning method. **D** Knockdown of p53, PLCL1 or SERPINB6. IMR5 cells stably transduced with either CHD5 expression or a mock control construct were treated for 48 h with siRNA against p53, PLCL1 or SERPINB6. Cells were analyzed by Western Blot for PLCL1, SERPINB6, and p53 expression. Shown is one representative experiment of three independent siRNA transductions. **E** Downregulation of PLCL1 and p53 but not SERPINB6 in CHD5-overexpressing cells enhances invasion and migration. Transduced and siRNA-treated cells were labeled with DiI and added to matrigel-coated transwell chambers. The top of the chamber contained medium with 1% serum and the lower part medium with 10% serum. 12 h post seeding, cells that had migrated through the matrigel and membrane, and were attached to the lower surface of the membrane, were fixed. The number of invasive cells per visual field (×20 magnification) is depicted in the graph. Means and SD are shown. Statistical analysis was performed using the *t* test. *p < 0.05, (*n* = 3 in duplicates).

To start to delineate potential target genes of CHD5 that may inhibit metastasis, genes expressed differentially between CHD5-overexpressing and control IMR5 cells, as determined by genome-wide mRNA sequencing, were assessed. 52 significantly differentially expressed genes were identified (Fig. 7A and Supplementary Table 1). For further analysis two candidates were selected from this list. First, PLCL1, as this protein is known to be involved in inhibition of tumor growth [38] and metastasis [39]. Second, SERPINB6, as serpin activity was one gene ontology class enriched in GSEA, and because SERPINB6 [40] inhibits metastasis and tumor progression. Given the proposed role of p53 in mediating the tumor-suppressive action of CHD5, p53 was also included in the analysis, although it was not differentially expressed.

As a first step, these genes were analyzed in three clinically annotated NB datasets within the R2 genomics analysis and visualization platform (http://r2.amc.nl) (Fig. 7B and Supplementary Fig. 2A). To assess, whether the candidate genes are independent of *MYCN*, the analysis was also performed in the *MYCN* non-amplified subsets of the datasets. SERPINB6 and p53 lose significance when *MYCN*-amplified tumors are excluded (Fig. 7B and Supplementary Fig. 2A). In contrast, high PLCL1 expression remains significantly associated with nonmetastatic disease independent of *MYCN* status (Fig. 7B and Supplementary Fig. 2A). In line, high PLCL1 expression is significantly associated with good overall survival in *MYCN* non-amplified tumors while SERPINB6 and p53 does not correlate in any of the datasets with patient outcome (Fig. 7C and Supplementary Fig. 2B).

To directly determine the effects of PLCL1, SERPINB6 and p53 on CHD5-mediated inhibition of metastatic mechanisms, IMR5 cells were treated with siRNA against those genes. Knockdown was confirmed at the protein level (Fig. 7D). In CHD5-overexpressing cells knockdown of PLCL1 and p53, but not SERPINB6, led to increased capacity to invade and migrate in extracellular matrix (Fig. 7E). In summary, PLCL1 appears to contribute to the metastasis-inhibiting effect of CHD5 in vitro and in patients, and p53 augmented CHD5-associated inhibition of invasion and migration of IMR5 cells in vitro.

## Discussion

*CHD5* is frequently lost and silenced in high-risk NB. This work provides evidence that CHD5 inhibits metastasis in this disease. Analysis of patient cohorts confirmed that low CHD5 expression in NB is associated with stage 4 metastatic disease and decreased survival. Low CHD5 expression is not associated with stage 4S, suggesting that genes other than *CHD5* may also control metastasis, and that the impact of CHD5 on prognosis is not solely due to an effect on metastasis. In vitro experiments validated that CHD5 has a tumor-suppressive function in NB cells. Importantly, CHD5 acts as a metastasis inhibitor in NB cells, independent of its tumor suppressor function. Several lines of evidence in vitro, in vivo and in silico support this notion. Anoikis resistance was decreased in NB cells with high expression of CHD5. Thus, fewer cells would presumably survive in the blood stream during hematogenous dissemination, leading to decreased colonization of target organs. Adherence to the endothelial cells lining the vessels of target organs, and invasion into and migration through the matrix of these organs are key steps in late hematogenous metastasis. Cells expressing CHD5 were less able to perform these tasks in vitro. Along this line, fewer NB cells expressing CHD5 homed to distant organs after i.v. injection into mice. Complementary long-term experiments in mice revealed that CHD5 expression increased latency and decreased incidence of metastases in bone marrow and liver, predilection sites of NB metastases in patients.

Interestingly, forced expression of CHD5 in SH-SY5Y cells, which have not lost 1p, did not decrease proliferation, clonogenic growth, anchorage-independent growth, adhesion to endothelial cells, invasion or migration in vitro. This is consistent with the notion that the tumor-suppressive effect of CHD5 in NB is confined to cells with 1p deletion [36].

In mice, the tumor-suppressive effect of CHD5 proceeds through the p19^ARF^–p53 axis [37]. We show in vitro that CHD5 does only to some extent decrease the aggressiveness of p53-mutant SK-N-BE(2)C cells (derived from relapse) or p53-depleted IMR5 cells. This strongly supports the conclusion that p53 is required for the tumor- and metastasis-suppressive effect of CHD5 in NB. As a corollary, these results help to explain the paucity of p53 mutations in NB at time of diagnosis.

Looking beyond p53, none of the known CHD5 target genes provided a satisfactory explanation how CHD5-associated metastasis inhibition may be mediated [34, 41, 42], thus transcriptome analysis was performed. Gene sets associated with glucose metabolism were enriched in NB cells not expressing CHD5, indicating that those cells might cope better with changing and challenging nutrient availability encountered by NB cells during metastasis [43]. Along this line, it is tempting to speculate that CHD5 may inhibit genes involved in the early steps of aerobic glycolysis.

In CHD5-expressing cells gene sets associated with cell-cell adhesion, p53 regulation, inactivation of MAPK signaling and neuronal differentiation were enriched, all consistent with a less metastatic phenotype. The association of CHD5 with neuronal differentiation is intriguing and warrants further investigation, as CHD5 has been described to enhance terminal differentiation of normal neurons [44]. Along this line, undifferentiated sympathetic neuroblasts would be expected to migrate during normal embryogenesis, yet differentiated sympathetic neurons should not, so the effects of CHD5 on the metastatic cascade may partly reflect a secondary effect of neuronal differentiation that is impaired in CHD5-low NB cells.

Searching for specific genes upregulated in CHD5-expressing cells, several lines of evidence suggested SERPINB6 as a promising candidate for mediating CHD5-associated metastasis inhibition in NB. However, when SERPINB6 was knocked down in IMR5 cells with forced expression of CHD5, CHD5-induced inhibition of invasion and migration was not reversed. In addition, *MYCN* amplification frequently associates with CHD5 loss in NB, so that enhanced SERPINB6 expression could merely be an indirect biomarker of *MYCN* amplification. Indeed, when correcting for *MYCN* amplification, SERPINB6 expression lost statistical significance in overall survival. Together, these data suggest that SERPINB6 does not mediate the metastasis-suppressive function of CHD5 in NB.

Thus, an additional differentially expressed gene potentially mediating CHD5-associated metastasis was explored. Phospholipase C Line 1 (PLCL1) was chosen, as its published functions suggest a role in metastasis. PLCL1 is a catalytically inactive protein binding PI(4,5)P2, thus functioning as a decoy for PI3K, impairing PI3K/AKT signaling and reducing tumor growth [38]. Concomitantly, PLCL1 inhibits polymerization of actin via PI(3,4,5)P3-WAVE2, thus impairing cell migration [39]. In silico analysis of *MYCN* non-amplified NB (to assess PLCL1 function independent of *MYCN*) in different independent patient cohorts revealed decreased expression of PLCL1 in stage 4 disease, and decreased patient survival associated with low expression of this gene. Knockdown of PLCL1 in IMR5 cells with forced expression of CHD5 reversed CHD5-induced inhibition of invasion and migration in vitro. We therefore conclude that PLCL1 may contribute to mediate the metastasis-suppressive function of CHD5.

There are several potential limitations of this study. First, the effects of CHD5 on tumor suppression and metastasis suppression may overlap. However, to disentangle this overlap, isolated steps of the metastatic cascade were assessed in vitro. In addition, NB cells were injected i.v. in the in vivo studies to exclude that different take and growth rates of primary tumors and thus different numbers of metastasizing cells distort the results of the experiments. The obvious limitation of this approach is that the impact of CHD5 on the early steps of metastasis, dissociation of cells from the primary tumor and extravasation, could not be assessed in vivo. Another limitation is the immunodeficient mouse model, which allowed the use of human NB cells but precluded assessment of immunological factors on metastasis. In summary, CHD5 is a metastasis suppressor in NB, mediated in part by PLCL1 and p53. Given, that CHD5 as a chromodomain-helicase modulates expression of many genes, additional, yet to be defined genes may contribute to the metastasis-suppressive effect of CHD5 in NB. Further work to reactivate CHD5 expression or its mediators of metastasis suppression is warranted.

## Materials and methods

### Generation of stable cell lines

Plasmids pF CAG luc puro (Addgene, Watertown, MA; Cat# 67501), precision LentiORF RFP control (PerkinElmer, Waltham, MA; Cat# OHS5832) and precision LentiORF CHD5 (PerkinElmer; Cat# OHS5898-202623270) were used to generate lentiviral particles in LentiX cells by using Lipofectamine LTX Reagent with PLUS Reagent (Thermo Fisher Scientific, Waltham, MA; Cat# 15338100) according to the manufacturer’s protocol. IMR5 and SK-N-BE(2)C cells were transfected with pF CAG luc puro and stably selected using 0.15–2.75 µg/ml puromycin (Sigma Aldrich, St. Louis, MO). Subsequently, IMR5, SK-N-BE(2)C, SH-SY5Y and GI-M-EN cells were transfected with MOI 5 of either LentiORF RFP control (mock) or LentiORF CHD5 without stop codon (CHD5), and stably selected using 4 µg/ml (SH-SY5Y and GI-M-EN), 9.75 µg/ml (IMR5), or 13 µg/ml (SK-N-BE(2)C) blasticidin (InvivoGen, San Diego, CA).

All cell lines have been authenticated by short tandem repeat profiles using the GenePrint 10 System (Promega, Mannheim, Germany) according to the manufacturer’s protocol. Cells were routinely tested and found to be negative for mycoplasma using the LookOut^®^ Mycoplasma PCR Detection Kit (Sigma Aldrich, Cat# MP0035-1KT) according to the manufacturer’s recommendations.

### Knockdown of genes by siRNA

NB cells were transiently transfected with siRNA at 20 µM (esiRNA, Sigma Aldrich; Cat# EHURLUC-20UG, EHU087001-20UG, EHU143431-20UG and EHU123221-20UG) using Lipofectamine RNAiMAX (Invitrogen, Carlsbad, CA) according to the manufacturer’s protocol. Downstream experiments were performed 48 h post transfection.

### Quantitative real-time PCR

qRT-PCR was performed as previously described [45] using the CFX ConnectTM Real-Time system (BioRad, Hercules, CA). For amplification, the following primers were used: CHD5 forward 5′-tgcttaaaggagcccaagtcc-3′, reverse 5′-tggtcagcgtgtggtaatcc-3′ and HPRT forward 5′-cctggcgtcgtgattagtga-3′, reverse 5′-cgagcaagacgttcagtcct-3′. mRNA expression levels were determined relative to HPRT expression.

### Adhesion assay on HUVEC

HUVEC were grown to monolayers in 24-well plates at standard conditions. NB cells were stained with 6.4 mg/ml 1,1′-dioctadecyl-3,3,3′,3′-tetramethyl-indocarbocyanine perchlorate (DiI, Thermo Fisher Scientific) and 5 × 10^4^ cells/ml were seeded per well on top of the HUVEC monolayer. After 1 h incubation at standard conditions, cells were washed twice before fixing with 3.7% paraformaldehyde. DiI-labeled cells in 12 visual fields were counted using a Keyence BZ-9000 microscope.

### Transwell invasion assay

Single cells were stained with 10 mg/ml DiI and 5 × 10^4^ (IMR5, GI-M-EN, SH-SY5Y) or 2.5 × 10^4^ (SK-N-BE(2)C) cells were seeded in medium with 1% FBS on top of matrigel-coated PET membranes with 8 µm pores (Corning, Corning, NY) placed in companion plates (Corning). The lower chamber was filled with medium containing 10% FCS. Cells were fixed 12 h (IMR5) or 20 h (SK-N-BE(2)C, GI-M-EN, SH-SY5Y) post seeding with 3.7% paraformaldehyde. Membranes were cut out and embedded. Cells, which had migrated through the membrane, were analyzed in 12 visual fields using a Keyence BZ-9000 micoscope.

### Animal experiments

To analyze initial homing, 5 × 10^6^ IMR5 cells were stained with 10 mg/ml DiI, suspended in 200 µl PBS and injected in the tail veins of 6–8 weeks old RAG2^−/−^cγc^−/−^ mice (*n* = 5 per group). Mice were sacrificed 2 h post injection and organs were collected for cryosectioning. 30 slides per organ were prepared and stained with Hoechst 33342 (1:1000; Thermo Fisher Scientific). Bone marrow samples were analyzed by flow cytometry using an Attune NxT cytometer (Thermo Fisher Scientific) and FlowJo 10.5.3 software (RRID:SCR_008520, Tree Star, Olten, Switzerland).

For analysis of metastases formation, 1 × 10^6^ cells were injected intravenously in 6–8 weeks old RAG2^−/−^cγc^−/−^ mice (*n* = 15 per group, female and male mice were equally assigned to both groups). Mice were analyzed by bioluminescence imaging once per week (Xenogen, IVIS 200, Caliper Life Sciences, Mainz, Germany). Four weeks post injection, mice were sacrificed and tissues were collected for histological staining. All experiments were performed according to institutional and state guidelines for the care and protection of animals.

### mRNA sequencing and bioinformatics analyses

Total RNA was isolated as described in Supplementary Materials and methods. Random-primed cDNA library preparation and stranded mRNA-Seq (NextSeq 500, 1 × 75 bp, 30 million reads, Illumina, San Diego, CA) of three samples per condition were performed by the EMBL Genomics Core Facility (Heidelberg, Germany). Reads were mapped to the human hg38 reference genome using bowtie2 (version 2.3.5.1, RRID:SCR_016368), assembled into putative transcripts and annotated. Average alignment rate was 87.2% (86.99–87.51%). Aligned files were sorted using samtools sort (version 1.9, RRID:SCR_002105) and read counting was performed using htseq-count from htseq (version 0.9.1). Differential expression analysis was performed using the R/Bioconductor DESeq2 (version 1.28.1, RRID:SCR_015687) package. Gene Set Enrichment Analysis (GSEA) was conducted by mapping results to the GO database (Molecular Signature Database (MSigDB); R-package msigdbr version 7.2.1) using the R/fGSEA (version 1.14.0) package. Data can be accessed via GSE183824.

### Statistical analysis

Data were analyzed using GraphPad Prism 8.4.3 software (RRID:SCR_002798, GraphPad Software, San Diego, CA) and are presented as the mean ± SD. For in vitro analyses no prespecified effect size was required. Three independent experiments were performed, unless stated otherwise. Appropriate sample size for animal experiments was determined by biometric evaluation. The unpaired, two-sided Student’s *t* test was used for comparison of two groups. One-way ANOVA was used for multiple group comparisons. Variance between groups was checked and found to be sufficiently similar. The log‐rank (Mantel–Cox) test was used for survival studies. Results were considered significant if *p* < 0.05.

Additional information can be found in Supplementary Materials and methods.

## Supplementary information

Supplementary Material
