# Supplementary material for: CHD5 inhibits metastasis of neuroblastoma
Source: Oncogene. 2021 Nov 17;41(5):622–33. doi: 10.1038/s41388-021-02081-0 (PMC8799470; doi:10.1038/s41388-021-02081-0)
Supplement: Supplementary file 1 — Supplementary Material [file 41388_2021_2081_MOESM1_ESM.pdf]

Supplementary Information

Supplementary Materials and Methods

Cell culture

The human neuroblastoma cell lines SK-N-BE(2)C, was purchased from ATCC (Cat# CRL-2268, Wesel, Germany) and cultured as described (1). SH-SY5Y and GI-M-EN cells were purchased from DMSZ (Cat# ACC 209 and ACC 654, Braunschweig, Germany) and cultured as described (1).The cell line IMR5 was a gift from G. M. Brodeur (CHOP, Philadelphia, PA) and cultured in RPMI-1640 (Invitrogen, Carlsbad, CA) supplemented with 20% heat-inactivated fetal bovine serum (FBS; Biochrom Ltd, Cambridge, UK), 2 mM L-glutamine and 100 U/mL penicillin/streptomycin (Invitrogen) in an atmosphere of 5% CO_2 at 37°C. Human umbilical vein endothelial cells (HUVEC) were purchased from Sigma Aldrich (St. Louis, MO, Cat# 200-05N) and cultivated as previously described (2). LentiX cells were purchased from TaKaRa Bio Inc. (Kusatsu, Japan; Cat# 632180) and cultured in DMEM (Invitrogen) supplemented with 10% heat-inactivated FBS, 2mM L-glutamine and 100 U/mL penicillin/streptomycin in an atmosphere of 5% CO_2 at 37°C.

Western blot analysis

Western blots were performed as previously described (1). Membranes were probed with the primary antibodies: anti-CHD5 (Cat# 44829, 1:500; Cell Signaling Technology, Danvers, MA), and anti-GAPDH (Cat# 5G4 Mab 6C5 1:8000; HyTest Ldt., Turku, Finland), anti-PLCL1 (Cat# ab157200,1:2500; abcam, Cambridge, United Kingdom), anti-SERPINB6 (Cat# TA504055, 1:500; OriGene, Rockville, Maryland) and anti-p53 (Cat# AH00152, 1:2500; Invitrogen). For secondary antibodies HRP-labeled goat anti-

rabbit IgG (Cat# 65-6120, 1:2500-1:10000; Invitrogen) and goat anti-mouse (Cat# 170-6516, 1:15000; BioRad, Hercules, CA) were used.

Images were taken with the ChemiDoc MP Imaging System (BioRad) using the Image Lab analysis software (BioRad).

Clonogenic growth assay

A single cell suspension of transfected NB cells was seeded in clonal density (7.5 x 10^2 cells/well) in 6-well plates in complete medium with blasticidin. Blasticidin was added every second day and cells were incubated at standard conditions for 10 – 14 days. Colonies were stained with 3.7% paraformaldehyde-laced crystal violet and counted using Fiji (3).

Metabolic activity assay

MTT (Sigma Aldrich) metabolic activity assays were performed as described (4). Briefly 2 x 10^3 (GI-M-EN), 4 x 10^3 (SK-N-BE(2)C) or 6 x 10^3 (IMR5 and SH-SY5Y) cells were seeded in 100 µl complete medium containing blasticidin in 96 well plates. Results were calculated relative to day 1 from at least 5 wells per experimental condition.

Soft agar clonogenicity assay

Single cell suspensions of 1 x 10^3 (IMR5, SK-N-BE(2)C) or 2 x 10^3 (SH-SY5Y) cells/ml were prepared in 0.5% low melting point agarose (LMP agarose, Sigma Aldrich) diluted in the respective cell line medium and seeded in 24-well plates. Growth medium containing blasticidin was replaced twice a week until colonies became visible. For analysis, colonies were stained with 1 mg/ml MTT and counted using Fiji.

Immunohistochemistry

Immunohistochemistry of formalin-fixed, paraffin-embedded sections from 5 mice per group was performed using standard protocols. Briefly, sections were deparaffinized, pressure-cooked or microwaved in citrate buffer, permeabilized with 0.1% Triton-X-100 and blocked with 10% goat serum and avidin/biotin blocking reagent (DAKO, Waldbronn, Germany). A 1:50 dilution of mouse anti-human CD57 (Cat# CM164B; Biocare Medical, Pacheco, CA), 1:20 of rat anti-mouse CD31 (Cat# dia310; Dianova, Hamburg, Germany), 1:100 rabbit anti-human CHD5 (Cat# 44829; Cell Signaling Technology) or 1:200 of mouse anti-human Ki67 (Cat# M7240; DAKO) was applied overnight at 4°C. Slides were incubated with secondary antibody solution and a 1:750 dilution of streptavidin-AP (Dianova). The Dako REAL™ Detection System (DAKO) was used according to the manufacturer's protocol. CD31-positive tumor vessels were counted in at least five visual fields from two metastases at 200x magnification. The percentage of positively stained tumor cell nuclei was determined in four visual fields at 400x magnification. Stained samples were analyzed using Keyence BZ-9000 microscope.

Supplementary Data

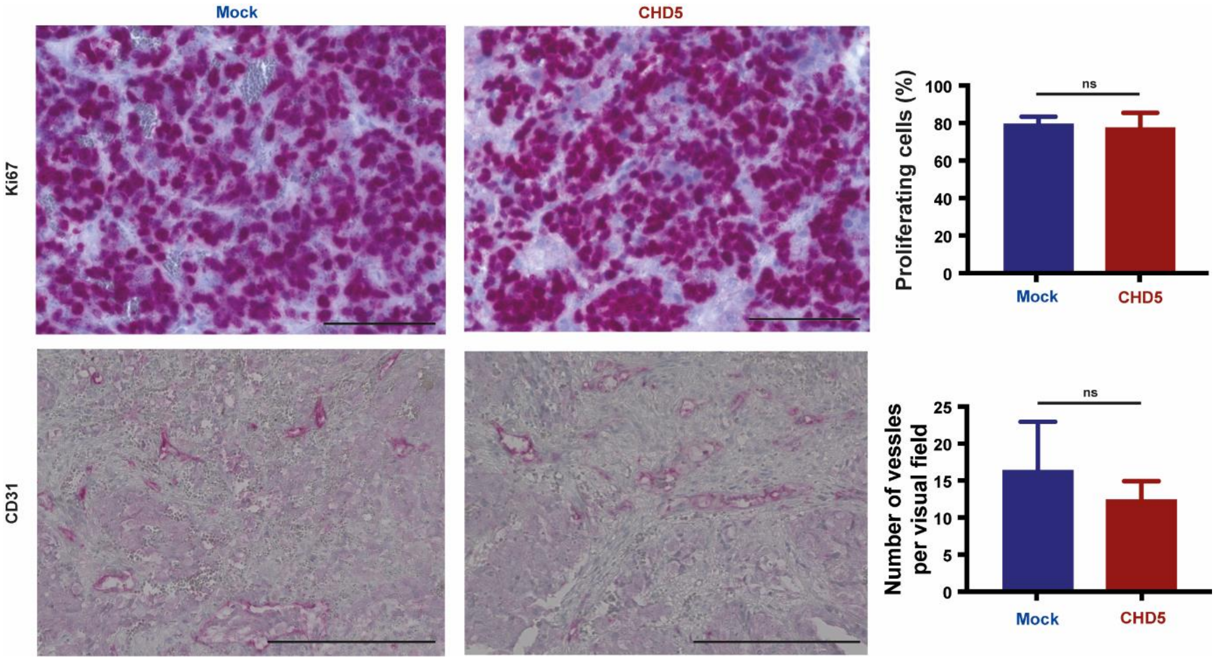

Suppl. Fig. 1: Forced expression of CHD5 does not alter proliferation and vascularization in the metastases.

Formalin-fixed, paraffin-embedded liver metastases from 5 mice per group sacrificed 28 d post cell injection were subjected to Ki67 and CD31 immunohistochemistry. At least 5 different visual fields from 2 metastases per mouse were analyzed. Representative pictures for each group are shown. Scale bar equals 100 µm. Means and SD are shown for each group. Statistical analysis was performed using t-test. ns, not significant.

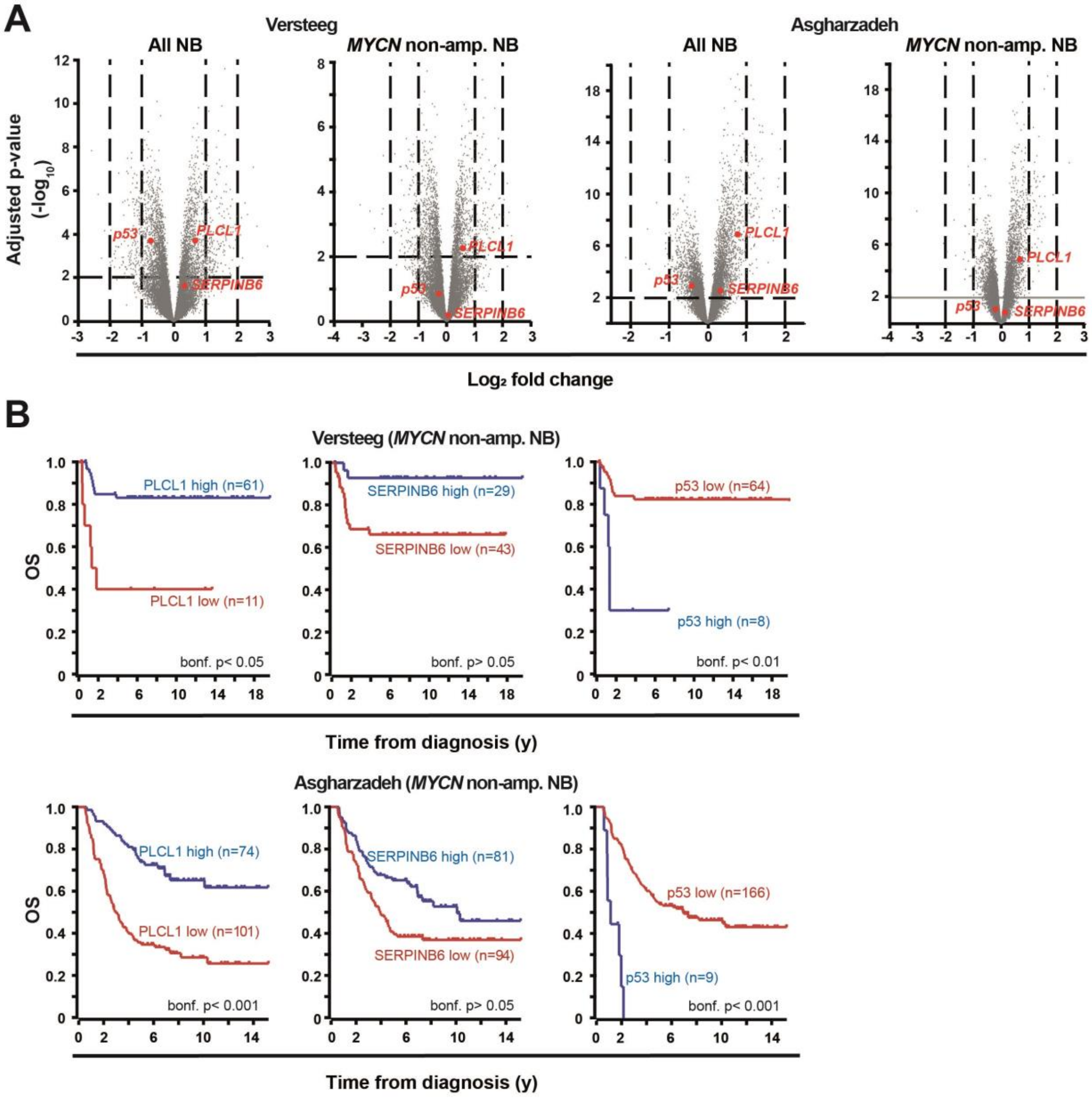

Suppl. Fig. 2: In additional data sets, too, PLCL1 contributes to the metastasis-inhibiting effect of CHD5 *in vitro* and *in situ*, and p53 *in vitro*.

A. High PLCL1 expression in patient NB is associated with non-metastatic disease. Gene expression depending on stage is shown in two different clinically annotated NB datasets (Asgharzadeh-249-custom-huex10t, n=247 and Versteeg GSE16476, n=76; R2 genomics analysis and visualization platform). Grey and red dots

in the volcano plot represent genes differentially expressed between stage 1-3 versus stage 4 patients.

B. High PLCL1 expression is associated with increased overall survival. Kaplan-Meier overall survival (OS) estimates for expression of PLCL1, SERPINB6 and p53 in *MYCN* non-amplified NB are shown. Results of the log-rank test are indicated. The cut-off was determined using the scanning method.

Suppl. Tab. 1: Differentially expressed genes

The 52 differentially expressed genes determined by mRNA sequencing in IMR5 cells overexpressing CHD5 and control cells based on log_2 fold change < - 0.2 and > 0.2 and p-adjusted < 0.05 are listed. Shown are Ensembl gene identifiers, gene names and corresponding statistics.

	Symbol	log_2 fold change	p-adjusted
ENSG00000116254	CHD5	6.230134664	7.64167E-49
ENSG00000136842	TMOD1	2.364281273	2.71404E-07
ENSG00000189067	LITAF	1.952290454	0.037884172
ENSG00000146592	CREB5	1.766958876	1.38087E-06
ENSG00000008283	CYB561	1.532253924	0.011159054
ENSG00000115896	PLCL1	1.38862969	0.019906759
ENSG00000274248	AJ011932.1	1.260513323	0.04376635
ENSG00000124570	SERPINB6	1.192143374	2.33849E-05
ENSG00000123240	OPTN	1.125703228	0.032590037
ENSG00000146216	TTBK1	1.122963256	0.037884172
ENSG00000139219	COL2A1	0.998727929	1.64569E-09
ENSG00000143507	DUSP10	0.865205772	0.002482005
ENSG00000141433	ADCYAP1	0.858139544	1.38087E-06
ENSG00000197106	SLC6A17	0.849081948	7.07908E-06
ENSG00000148357	HMCN2	0.829589163	2.33849E-05
ENSG00000157680	DGKI	0.81291283	0.012306726
ENSG00000177606	JUN	0.768073061	1.2035E-07
ENSG00000184785	SMIM10	0.701760062	0.015728091
ENSG00000054690	PLEKHH1	0.686098205	0.018870801
ENSG00000099250	NRP1	0.636061911	6.5852E-06
ENSG00000132932	ATP8A2	0.633365392	0.04101218
ENSG00000244300	GATA2-AS1	0.625575596	0.014997842
ENSG00000180537	RNF182	0.613522431	0.00291682
ENSG00000189056	RELN	0.57470146	0.002482005
ENSG00000135549	PKIB	0.557194895	0.008904255
ENSG00000164484	TMEM200A	0.534469578	0.037884172
ENSG00000176244	ACBD7	0.52152602	0.020862063
ENSG00000151690	MFSD6	0.50979967	0.037884172
ENSG00000091409	ITGA6	0.481304804	0.019906759
ENSG00000102362	SYTL4	0.47111895	0.011643571
ENSG00000149257	SERPINH1	0.283722748	0.026258044
ENSG00000038427	VCAN	-0.290533786	0.036059219
ENSG00000145901	TNIP1	-0.349385355	0.032569478
ENSG00000091947	TMEM101	-0.361884248	0.036059219
ENSG00000134871	COL4A2	-0.394299113	0.034735636
ENSG00000162599	NFIA	-0.433319098	0.032797045
ENSG00000185070	FLRT2	-0.439240516	0.011643571

ENSG00000162998	FRZB	-0.450858832	0.049330907
ENSG00000181418	DDN	-0.45884312	0.019906759
ENSG00000101115	SALL4	-0.477171463	0.049929502
ENSG00000065989	PDE4A	-0.537505133	0.014737817
ENSG00000173068	BNC2	-0.560631079	0.02642434
ENSG00000184226	PCDH9	-0.563992227	0.011643571
ENSG00000145808	ADAMTS19	-0.619297173	0.032590037
ENSG00000152284	TCF7L1	-0.636128798	0.027078037
ENSG00000174498	IGDCC3	-0.661871887	9.58532E-09
ENSG00000140937	CDH11	-0.669365983	0.001146073
ENSG00000169946	ZFPM2	-0.679020625	0.003380899
ENSG00000134853	PDGFRA	-0.739844789	5.68551E-06
ENSG00000188729	OSTN	-0.91115143	0.002482005
ENSG00000115461	IGFBP5	-1.117367211	0.001364205
ENSG00000167680	SEMA6B	-1.242799993	3.07198E-13

Supplementary References

1. Dorneburg C, Fischer M, Barth TFE, Mueller-Klieser W, Hero B, Gecht J, et al. LDHA in Neuroblastoma Is Associated with Poor Outcome and Its Depletion Decreases Neuroblastoma Growth Independent of Aerobic Glycolysis. Clin Cancer Res. 2018 Nov 15;24(22):5772–83.

2. Schlitter A-M, Dorneburg C, Barth TFE, Wahl J, Schulte JH, Brüderlein S, et al. CD57high Neuroblastoma Cells Have Aggressive Attributes Ex Situ and an Undifferentiated Phenotype in Patients. Ulasov I, editor. PLoS ONE. 2012 Aug 10;7(8):e42025.

3. Schindelin J, Arganda-Carreras I, Frise E, Kaynig V, Longair M, Pietzsch T, et al. Fiji: an open-source platform for biological-image analysis. Nat Methods. 2012 Jul;9(7):676–82.

4. Mosmann T. Rapid colorimetric assay for cellular growth and survival: Application to proliferation and cytotoxicity assays. J Immunol Methods. 1983 Dec;65(1–2):55–63.
